# Molecular determinants of etoposide resistance in HL60 cells

**DOI:** 10.6026/97320630018894

**Published:** 2022-10-31

**Authors:** Rasha H Alghamdi, Farid Ahmed, Sara M Ibrahim, Peter N Pushparaj, Hans Jurgen Schulten, Adel M Abuzenadah, Abdulrahman L Almalki

**Affiliations:** 1 Center of Excellence in Genomic Medicine Research, King Abdulaziz University, P.O. Box 80216, Jeddah21589, Kingdom of Saudi Arabia; 2 Department of Biochemistry, Faculty of Science, King Abdulaziz University, P.O. Box 80218, Jeddah21589, Kingdom of Saudi Arabia; 3 Department of Chemistry, Faculty of Science, Taif University, P. O. Box 11099, Taif 21944, Kingdom of Saudi Arabia; 4 Faculty of Applied Medical Sciences, King Abdulaziz University, P.O. Box 80216, Jeddah, 21589, Kingdom of Saudi Arabia; 5 King Fahad Medical Research Center, King Abdulaziz University, P.O. Box 80216, Jeddah21589, Kingdom of Saudi Arabia

**Keywords:** Cancer, Chemotherapy, Multidrug Resistance, Etoposide, Apoptosis, Acute Myeloid Leukemia

## Abstract

Chemotherapy resistance is the main reason for treatment failure in acute myeloid leukemia (AML) and the major cause of its mortality. Etoposide is a DNA topoisomerase-II inhibitor that is used either as a single agent or in combination with cytarabine, azacytidine, vinca alkaloids, and anthracyclines for the treatment of relapsed /refractory AML. In this study, we sought to determine and understand the mechanism of etoposide resistance in AML using the HL60 cell line.HL60 cells were treated with incremental doses of etoposide and resistant colonies were isolated by culturing the resistant cells in semi-solid culture media. Three clones were selected for etoposide resistance namely, HL60-EtopR H1A, HL60-EtopR H1B, and HL60-EtopR H1C which demonstrated 4.78, 2.39, and 4.42-fold higher resistance to etoposide compared with the parental cells. To determine molecular differences between the etoposide-resistant HL60-EtopR cells and the parental cells, microarray-based gene expression profiling was performed. We found up regulation of members of the src tyrosine kinase family genes in the etoposide resistant cells. Further studies are required to evaluate the role of Src inhibitors in targeting etoposide resistant cells.

## Background:

Acute myeloid leukemia (AML) is the most common form of blood cancer in older adults with an average age of 65-70 years at diagnosis [1]. It is a heterogeneous and clonal disease of hemopoietic progenitor cells, characterized by their abnormal proliferation and impaired differentiation, leading to accumulation of immature myeloid cells in the peripheral blood and bone marrow. Treatment strategies for AML include chemotherapy, radiotherapy, and allogeneic bone marrow transplantation [2]. However, combination chemotherapy consisting of cytarabine (Ara C) and an anthracycline, has been the backbone of AML treatment for several decades [3]. Although complete remission (CR) is achieved in 40-60% of older AML (>60 years), majority (80-90%) of them eventually relapse [4]. Relapse and refractory AML have very poor outcomes. Etoposide (VP-16) is used either alone or in combination with mitoxantrone for relapsed/refractory AML [5-7]. Etoposide is derived from podophyllotoxin which is extracted from the rhizome of mayapple (Podophyllum peltatum). It inhibits DNA synthesis by forming a complex with topoisomerase II and DNA, resulting in increased double-stranded DNA breaks and the inability to repair DNA damage. Accumulation of damaged DNA results in cell cycle arrest and apoptosis [8]. Etoposide is an amphipathic compound that passively enters the cell membrane by flip flop mechanism and is a known substrate of ABC transporters [9].Acquired resistance to etoposide has been observed in multiple cancer cells and several mechanisms of resistance have been identified. These mechanisms include upregulation of the drug efflux pump ABCB1, down regulation of topoisomerase II gene expression [10], long non-coding satellite III RNA (Sat III) mediated recruitment of topoisomerase II to nuclear stress bodies [11], and mutations in gene encoding topoisomerase II [12]. In this study, we sought to generate and characterize etoposide resistant leukemic cell line and compare the gene expression profile with the sensitive parental cells to gain molecular insights into acquired etoposide resistance. Therefore it is of interest to understand the mechanism of etoposide resistance in the HL60 clones would facilitate the development of strategies to overcome resistance and restore responsiveness in the resistant cells.

## Methodology:

### Cell culture:

HL60 cells (CLS GmBH, Germany) were cultured in RPMI media (Gibco, Life Technologies, USA) supplemented with 10% FBS (Gibco) and maintained at 37°C in a humidified incubator. Etoposide resistant cells HL60-EtopRwere generated by culturing HL60 cells in gradually incremental doses of Etoposide (2nM to 1µM) over a period of 2 months.Single cell clones of etoposide resistant cells were isolated by culturing the drug selected cells in methylcellulose-based semisolid media and picking up isolated colonies of resistant cells.These isolated clones were named: HL60-EtopRH1A, HL60-EtopRH1B, and HL60-EtopRH1C.

### Cell viability assay:

Cell viability assay was performed using the CellTiter-Blue®assay (Promega) according to the manufacturer's instructions. Approximately 10,000 cells were counted and incubated with the drug dilutions in96-well plates at 37°C for 48h. CellTiter-Blue reagent was added and incubated for an additional 2h. Fluorescence was measured at 540Ex/590Em using SpectraMaxi3 Microplate Reader (Molecular Devices, USA). The mean inhibitory concentration of the drugs (IC50) was plotted using the non-linear regression model.

### Annexin V apoptosis assay:

HL60EtopR cells (H1A, H1B and H1C), and HL60control cells were counted at 1x105 cells/ml/well and plated in a 12-well plate. Etoposide was added to the cells at the indicated doses (50µM and 200µM) and incubated at 37°C for 48hours. At the end of incubation, cells were collected, washed twice in cold PBS, and then resuspended in 1xannexin binding buffer. Allophycocyanin (APC)-labeled Annexin V and 7AAD (BD Biosciences, USA) were added to the cells according to the manufacturers’ recommendations and stained for 15minutes in the dark at room temperature. Samples were then analyzed on BD-FACS Aria-III flow cytometer (BD Biosciences, USA).

### Microarray-based gene expression profile:

RNA from HL60 and HL60EtopRcells(H1A, H1B and H1C)was extracted using the Total RNA Prep Kit from BioFACT (BIOFACTORY, Korea), according to the manufacturer's recommendations.Microarray assay was performed using the Affymetrix (Santa Clara, CA) GeneChip™ Human Genome U133 Plus 2.0 arrays according to the manufacturer's instructions. GeneChip IVT Express kit was used to prepare of labelled cRNA that was subsequently amplified and fragmented. The components of GeneChip Hybridization, wash and stain were used for hybridization and evaluated in the GeneChip Human Genome U133 plus 2.0 arrays. The probe array of hybridization was placed in the GeneChip Hybridization Oven 640 at 45°C for 16±1hours. The arrays were then scanned using Affymetrix GeneChip® scanner 3000 7G with Affymetrix GeneChip Command Software (AGCC). Affymetrix CEL files were imported into Partek Genomic Suite version 6.6 (Partek Inc., MO, and USA). The Robust Multichip Average (RMA) was used to normalize analyzed data.Analysis of variance (ANOVA) was performed using p-values < 0.05 and cut off fold change (FC) ≥ 1.5, the two most common criteria for identifying differentially expressed genes.

### Statistical analysis:

All statistical analyses were performed using Microsoft Excel or GraphPad Prism software version 9.0 (GraphPad Software, Inc, USA).

## Results:

### Etoposide resistant HL60 cells demonstrated varying levels of resistance:

To verify the resistance in the etoposide resistant HL60 clones, cell viability experiment was performed by incubating the cells in etoposide (concentrations ranging from 0.01µM to 100µM) for 48hours and detected using CellTiter-Blue® assay. The mean inhibitory concentration causing 50% cell death, IC50, for the three clones of etoposide resistant HL60-EtopR H1A, HL60-EtopR H1B, and HL60-EtopR H1C were 4.16±2.11µM, 2.25±2.09µM and 3.38±1.68µM respectively as compared to the etoposide sensitive parental HL60 cell line, which displayed a relatively low IC50 of 0.86±0.34µµ as shown in Table 1. Therefore, the etoposide resistant HL60-EtopR H1A, HL60-EtopR H1B, and HL60-EtopR H1C showed4.78, 2.39 and 4.42-fold higher resistance respectively compared with the parental HL60 cells (Figure 1 - see PDF).

### Etoposide resistant HL60 cells demonstrate resistance to apoptosis:

Induction of apoptosis was assessed by Annexin V-APC-7AAD staining using flow cytometry in HL60-EtopRH1A, HL60-EtopRH1B, and HL60-EtopRH1C, cells treated with 50µM and 200µM Etop for 48hours. As shown in Figure 2 and 3(see PDF), Etop induced high percentage of apoptosis in the parental cells at 50µM that was subsequently increased at 200µM. The resistant clone's i.e.HL60-EtopRH1A, HL60-EtopRH2B and HL60-EtopRH3C did not show any significant apoptosis even at 200µM. Thus, it is evident that etoposide resistance is related to the decrease in cells undergoing apoptosis in the resistant clones compared with the parental cells.

### Etoposide resistant cell line show increased Src Tyrosine Kinase family in gene expression analysis:

To further demonstrate the molecular mechanism of Etop resistance, microarray-based gene expression profiling was performed for the parental HL60 and HL60-EtopR cells. Significant differences in gene transcripts were identified between three independent clones of HL60-EtopR cells and sensitive HL60 cells. Of all the differentially regulated gene transcripts, 1081 were upregulated and 812 were downregulated. The 40 highly regulated genes are described in Table 2(see PDF). No drug transporter belonging to the ABC family was found upregulated in the resistant cells. Interestingly, some members of the Src tyrosine kinase family gene transcript were found to be significantly upregulated. For example, HCK (12.05-fold) and FGR (9.64-fold) were upregulated in HL60-EtopR H1 cells compared with the parental HL60 cells. This highlights the role of Src tyrosine kinase family genes in the resistance observed in etoposide resistant HL60 cells.

## Discussion:

AML is a highly heterogeneous disease with a poor clinical prognosis and an overall survival rate of <30% [13].Despite tremendous advances in cancer treatment, chemotherapy remains the mainstay of AML treatment. However, there has been a dramatic shift in the clinical scenario over the past three years with the rapid approval of 8 novel agents for different indications of AML [14].The success of chemotherapy is critically dampened by the development of resistance. Even with the most favorable prevailing therapeutic options, resistance remains a major obstacle to achieving complete remission and patients initially responding to treatment may relapse with a more vigorous form of the disease, insensitive to the cytotoxic effect of drugs used. Studying acquired resistance of human cancer cells to chemotherapeutic agents is pivotal in understanding the molecular complexities of cancer and to find alternative treatment approach that could circumvent chemotherapeutic resistance. Etoposideeither alone or with mitoxantrone has been used to treat relapsed or refractory AML [5-7]. Up regulation of ATP-binding cassette (ABC) transporter super family proteins such as ABCB1, ABCG2 and ABCC1 which pump chemotherapeutic drugs out of tumor cells thereby reducing intracellular drug accumulation have been observed as the most common mechanism of chemotherapy resistance [15]. Leukemic cell lines resistant to adriamycin and over expressing ABCB1 demonstrate co-resistance to etoposide [16]. However, our leukemia cell line model selected for etoposide resistance, did not demonstrate upregulation of ABC transporter proteins. Another majorly known mechanism of etoposide resistance is the altered expression of its target enzyme, the topoisomerase II (Topo II) [17]. We did not observe any change in the Topo II expression at the mRNA level. We observed upregulation of the SRC kinase family proto-oncogenes HCK and FGR (12.05 and 9.64-fold respectively). Activation of Src kinase has previously been shown to inhibit apoptosis induction by etoposide [18]. Src is a non-receptor tyrosine kinase that exerts molecular control over various aspects of neoplasticity. Hck, and Lyn are members of the Src kinase family that are frequently over expressedin AML leukemic stem cells.

Up regulation of Hck was detected in leukemic stem cells from AML patients who relapsed after chemotherapy. Interestingly, inhibition of Src completely restored the chemo sensitivity of primary cells when engrafted in mice [19]. Similarly, several studies have reported that Fgr is over expressed in AML; its suppression resulted in decreased growth of primary cells [19,20]. These studies are concordant to our findings and provide an important target for reversing etoposide resistance in HL60 clones. Xylosyltransferase (XYLT1) is a type II membrane protein and a negative regulator of the Notch receptor. Although activation of the Notch pathway has been implicated in oncogenesis, its exact role in AML is unclear. One study reported decreased expression of its downstream targets in AML, with activation of Notch inhibiting AML growth and survival [21]. However, another study showed that reduced Notch activity together with Wnt activation was associated with the remodeling of myeloid progenitor cells in mouse model under stress [22]. This is highly relevant to our findings as a 25.5-fold increase of XYLT1 in HL60 R clones reflects down regulation of Notch signaling, potentially resulting in the cellular reprogramming of leukemic stem cells to combat etoposide cytotoxicity. Further work is required to confirm Notch down regulation and clarify the role of Notch signaling in etoposide resistance in AML Enzymes of amino acid metabolism such as ornithine amino transferase (OAT) are emerging as important therapeutic targets that work by disrupting the metabolic machinery of cancer cells and depriving them of essential nutrients for their growth and survival. Up regulation of OAT has been significantly associated with hepatocellular carcinoma development with inhibition of these enzymes, showing promising results in reducing tumor [23]. In our study, etoposide resistant HL60 clones showed 65-fold down regulation of OAT. Thus, the role of OAT in chemo resistance remains elusive, as it has a specific survival advantage in AML-resistant cells.HL60 R clones also showed a sharp decrease in mRNA levels of helicase-like transcription factor (HLTF). This could be of immense clinical importance, as decreased HLTF expression in the bone marrow of AML patients has been associated with unfavourable prognosis and disease progression. Thus, HLTF may play a potentially chemo protective role in AML that remains to be explored [24]. Long non coding RNAs (ln RNAs) are small RNA molecules (<200 nucleotides) that regulate various cellular functions by interfering with gene transcription. Several ln RNAs have been shown to play an important role in the development of the MDR phenotype in breast cancer [25]. Specifically, LINCO1215 was found to be upregulated in ovarian cancer andits downregulation decreased tumor growth and metastasis [26]. Surprisingly, we observed that LINC01215 expression wasreducedby 12-folds in the resistant clones.

## Conclusions:

In summary, we have identified several differentially expressed genes in acquired resistance to etoposide in the HL60 cells. Our results demonstrate overexpression of several members of the Src Kinase family indicating their possible role in etoposide resistance. Further functional assays will help to understand the mechanism of etoposide resistance in leukemia cell lines and confirm the role of some of the key molecular targets found in this study. Understanding the mechanism of etoposide resistance in HL60 clones would facilitate the development of strategies to overcome resistance and restore cell responsiveness.
